# Cucurbitacin B Exhibits Antitumor Effects on Chordoma Cells via Disruption of Brachyury

**DOI:** 10.3390/ijms26083864

**Published:** 2025-04-18

**Authors:** Carolin Seeling, Johannes Neumahr, Fabian Häberle, André Lechel, Peter Möller, Nadine T. Gaisa, Thomas F. E. Barth, Kevin Mellert

**Affiliations:** 1 Institute of Pathology, University Hospital Ulm, Albert-Einstein-Allee 11, 89081 Ulm, Germany; 2 Department of Internal Medicine III, University Hospital Ulm, 89081 Ulm, Germany; 3 Department of Internal Medicine I, University Hospital Ulm, 89081 Ulm, Germany

**Keywords:** chordoma, brachyury, Cucurbitacin B, cytoskeleton, protein unfolding

## Abstract

Chordomas are rare malignant tumors of the bone, originating from remnants of notochordal cells. The transcription factor brachyury, encoded by *TBXT*, serves as a critical diagnostic marker and is essential for tumor growth. While brachyury’s role in regulating the cytoskeleton during embryogenesis and tumorigenesis is well understood, the reverse—whether cytoskeletal alterations can influence brachyury levels—remains unclear. Despite advances in understanding chordoma biology, there are currently no approved targeted therapies, underscoring the need for novel therapeutic approaches. Three chordoma cell lines were treated with cytoskeletal inhibitors, including the actin-targeting compounds Cucurbitacin B (CuB) and Latrunculin B (LatB). Morphological changes, *TBXT* expression, and cell viability were analyzed. The effects of CuB were examined over time and across concentrations, with cell viability assessed via apoptosis and cytotoxicity assays. Microarray gene expression profiling of ten chordoma cell lines was performed to explore CuB-mediated transcriptional changes. Rescue experiments using a *TBXT* open reading frame vector and co-treatments with autophagy and proteasome inhibitors were conducted to elucidate the mechanisms of brachyury depletion. Both CuB and LatB induced significant morphological changes, but only CuB caused near-complete depletion of brachyury. This effect was time- and concentration-dependent, correlating with reduced cell viability driven primarily by apoptosis. Microarray analysis revealed that CuB treatment upregulated protein refolding pathways and downregulated protein glycosylation. Notably, *TBXT* transcription was only slightly suppressed, indicating that brachyury depletion was largely post-transcriptional. Rescue experiments and co-treatments implicated dysregulated protein refolding and endoplasmic reticulum (ER) stress as key mechanisms underlying CuB-mediated brachyury loss. This study demonstrates that actin cytoskeleton disruption by CuB depletes brachyury in chordoma cells, primarily through dysregulated protein refolding and ER stress rather than transcriptional repression. These findings suggest that targeting actin cytoskeleton dynamics or protein unfolding pathways may provide novel therapeutic approaches for chordoma treatment.

## 1. Introduction

Chordomas are rare malignant neoplasms believed to arise from remnants of the notochord, accounting for approximately 1–4% of all primary bone tumors. These tumors predominantly occur along the axial skeleton, with a marked prevalence in the sacral region and at the skull base [1,2]. The typical age of onset for chordomas is in the sixth decade [3].

Although chordomas grow slowly, they exhibit significant local aggressiveness and have recurrence rates that can exceed 50%. Metastatic spread is observed in approximately 20% of patients, primarily affecting the lungs, bones, and soft tissues [4,5]. Current standard treatment modalities for chordoma include surgical resection, with or without adjuvant radiotherapy, and definitive radiation for unresectable tumors. However, achieving en bloc resection with negative margins is often challenging due to its close proximity to critical neural structures [6].

Recent progress in molecular characterization of chordomas has facilitated the exploration of systemic therapeutic strategies for patients who are not candidates for surgical intervention or radiotherapy. Targeted therapies under investigation include tyrosine kinase inhibitors such as Imatinib and Dasatinib (targeting PDGFR and KIT), Erlotinib, Afatinib, Cetuximab, and Lapatinib (targeting EGFR and Her-2-neu), in addition to VEGFR inhibitors like Sorafenib and Pazopanib [7,8,9,10,11,12,13]. Furthermore, recurrent genetic alterations, including copy number loss of *CDKN2A* and deletions of the *PTEN* locus, support the rationale for employing pathway-specific inhibitors, such as Palbociclib (targeting CDK4/6) and Sirolimus (targeting mTOR) [14,15,16,17,18]. Despite these therapeutic advancements, no pharmacological agents have yet received approval for the treatment of chordoma, and significant efficacy of experimental therapies remains to be established.

At the molecular level, brachyury, a transcription factor encoded by the *TBXT* gene, has emerged as a pivotal biomarker and a potential therapeutic target in chordoma [19]. During embryogenesis, brachyury is well-established as a regulator of mesodermal formation and the differentiation of mesenchymal cells and is essential for the development of the notochord. Its role in embryonic development extends to orchestrating the epithelial-to-mesenchymal transition (EMT) [20].

In chordomas, brachyury plays a crucial role in maintaining the identity and survival of chordoma cells, underscoring its dual role as both a diagnostic marker and a driver of tumor cell identity [19,21]. The experimental knockdown of *TBXT* in chordoma cell lines results in marked alterations in cellular morphology, including growth arrest and a transition from the characteristic vacuolated cell structure to a spindle-shaped phenotype. This phenotypic shift suggests that, in the absence of brachyury, chordoma cells may revert to a more primitive, mesenchymal-like state, emphasizing the deep connection between developmental biology and tumor cell behavior.

This morphological shift closely resembles EMT-like transitions observed during embryonic development, where cytoskeletal reorganization enables cells to become more elongated and motile [22,23].

These findings raise the intriguing possibility of a reciprocal relationship between brachyury and cytoskeletal dynamics. While brachyury’s influence on cytoskeletal organization is well elucidated, it remains to be explored whether changes in the cytoskeleton might, conversely, regulate brachyury expression, potentially influencing tumor progression.

Therefore, this study aims to investigate whether cytoskeletal changes might influence brachyury levels in chordoma cells, drawing on parallels from embryological processes where cytoskeletal dynamics and transcription factor networks are closely intertwined.

## 2. Results

### 2.1. Evaluation of Cytoskeletal Inhibitors on Brachyury Levels in Chordoma Cells

To investigate the effects of various cytoskeletal inhibitors on brachyury levels, the chordoma cell line U-CH1 was treated for 48 h with CuB, Latrunculin B (LatB), Narciclasine, Colchicine, and Vincristine, adopting IC_50_ values from the literature [24,25,26,27]. We oriented the concentrations at the upper end of the published ranges, as chordoma cells, known for their vacuolated cytoplasm, often require higher inhibitor concentrations to observe significant effects. Additionally, a 48 h incubation period was chosen to ensure adequate time for translational responses to occur downstream of cytoskeletal disruption, as shorter treatment durations may not allow these effects to manifest fully. This extended treatment period has been shown to effectively reduce protein levels in similar experimental setups using other compounds [10].

Equivalent volumes of DMSO or ddH_2_O were applied as negative controls to assess baseline conditions. To exclude nonspecific effects on global gene expression, protein levels of cytoplasmic and nuclear located housekeeper proteins (vinculin, β-actin, GAPDH, and histone H3) were measured.

**Figure 1:**
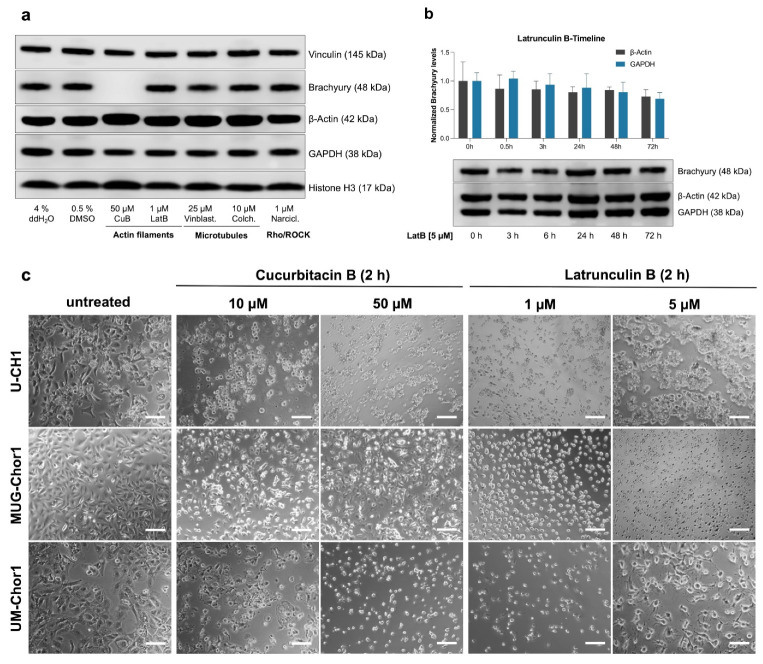
Effects of actin and microtubule inhibitors on brachyury levels and chordoma cell morphology. (**a**) Western blot analysis of brachyury and cellular markers in U-CH1 chordoma cells treated with 50 µM Cucurbitacin B (CuB), 1 µM Latrunculin B (LatB), 25 µM Vinblastine (Vinblast.), 10 µM Colchicine (Colch.), or 1 µM Narciclasine (Narcil.), compared to vehicle controls (4% ddH_2_O and 0.5% DMSO). CuB treatment caused nearly complete depletion of brachyury protein, whereas no significant changes in brachyury levels were observed with the other treatments. (**b**) Time course analysis of brachyury levels in U-CH1 cells treated with LatB (5 µM). Brachyury levels, normalized against β-actin or GAPDH to ensure accurate normalization, especially given potential actin perturbation. Data represent means ± SD from three independent experiments (*n* = 3). (**c**) Phase-contrast microscopy of U-CH1, MUG-Chor1, and UM-Chor1 chordoma cells after 2 h of treatment with CuB (10 µM or 50 µM) and LatB (1 µM or 5 µM). These concentrations were selected based on the results shown in panels (**a**,**b**). The 2 h time point was chosen to highlight early morphological changes. Both CuB and LatB induced pronounced cell rounding and detachment in all tested cell lines. Scale bar = 100 µm.

Treatment with Vinblastine and Colchicine, inhibitors of microtubule polymerization, did not elicit significant changes in cellular morphology or brachyury protein levels, as determined by Western blot analysis (Figure 1a). Similarly, Narciclasine, an inhibitor of the Rho/ROCK-signaling pathway, had no impact on either cellular morphology or brachyury expression.

In contrast, while both CuB and LatB disrupted actin polymerization, only CuB induced a pronounced reduction in brachyury protein levels, rendering it nearly undetectable following treatment. Notably, despite this disparity in brachyury regulation, both CuB and LatB caused comparable morphological alterations in U-CH1, MUG-Chor1, and UM-Chor1 cells, including detachment from the substrate and pronounced cell rounding (Figure 1c). Even at higher concentrations and prolonged exposure durations, LatB treatment had only minimal effect on brachyury levels in U-CH1 cells, as determined by Western blot analysis (Figure 1b). Unlike CuB, the cytoskeletal disruption induced by LatB appeared reversible, with chordoma cells beginning to re-adhere after 48 h of treatment (Supplementary Figure S1; Additional File S2). Given the profound impact of CuB on reducing brachyury, its potential as a therapeutic agent was further explored.

### 2.2. Cucurbitacin B Has Antiproliferative Effects in Chordoma Cell Lines

**Figure 2:**
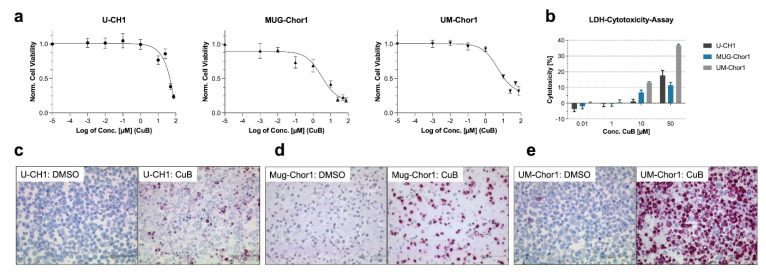
Cucurbitacin B (CuB) reduces cell viability by inducing apoptosis in chordoma cell lines. (**a**) Cell viability analysis by MTS assays following treatment of U-CH1, MUG-Chor1, and UM-Chor1 cell lines with increasing concentrations of CuB. CuB treatment resulted in a dose-dependent reduction in cell viability. (**b**) Cytotoxicity analysis using LDH assay in the same cell lines treated with varying concentrations of CuB. CuB induced a concentration-dependent increase in cytotoxicity. Error bars represent the standard deviation (SD), with experiments performed in triplicate (*n* = 3). Immunocytochemistry of the apoptosis marker cleaved caspase-3 in U-CH1 (**c**), MUG-Chor1 (**d**), and UM-Chor1 (**e**) cells treated for 48 h with DMSO or 50 µM CuB. CuB treatment led to a marked increase in cleaved caspase-3 staining, indicating apoptosis induction. Scale bar = 100 µm.

To evaluate the antiproliferative effects of CuB, the chordoma cell lines U-CH1, MUG-Chor1, and UM-Chor1 were treated with increasing concentrations of CuB for 48 h. A significant reduction in cell viability to less than 30% was observed in all cell lines. The calculated IC_50_ values were 3.0 µM ± 0.85 µM for MUG-Chor1, 5.1 µM ± 0.81 µM for UM-Chor1, and 60.0 µM ± 4.3 µM for U-CH1 (Figure 2a).

As the MTS assay reflects overall metabolic activity, it captures both growth inhibition and cell death but cannot distinguish between the two. To specifically assess cytotoxicity, LDH assays were performed, which measure the release of LDH from damaged (typically necrotic or late apoptotic cells), offering a more precise indication of membrane integrity and cellular damage leading to cell death (Figure 2b). Initial cytotoxic effects were detected at 10 µM CuB, with 1.2% ± 1.1%, 6.9% ± 1.2%, and 13.2% ± 0.4% of cells showing signs of cytotoxicity in U-CH1, MUG-Chor1, and UM-Chor1, respectively. At 50 µM CuB, cytotoxicity increased to 17.7% ± 2.5% in U-CH1, 11.5% ± 1.5% in MUG-Chor1, and 36.8% ± 0.6% in UM-Chor1.

Despite these cytotoxic effects, the magnitude of cytotoxicity did not fully account for the substantial reduction in cell viability observed in the MTS assays. To further investigate the underlying mechanism, immunocytochemical staining for cleaved caspase-3 was performed, revealing a high proportion of apoptotic cells in U-CH1, MUG-Chor1, and UM-Chor1 after 48 h of treatment with 50 µM CuB (Figure 2c–e). These findings indicate that the observed reduction in cell viability is primarily due to early apoptotic effects, rather than direct cytotoxicity.

### 2.3. Dose-Dependent Reduction in Brachyury Protein in Chordoma Cell Lines

**Figure 3:**
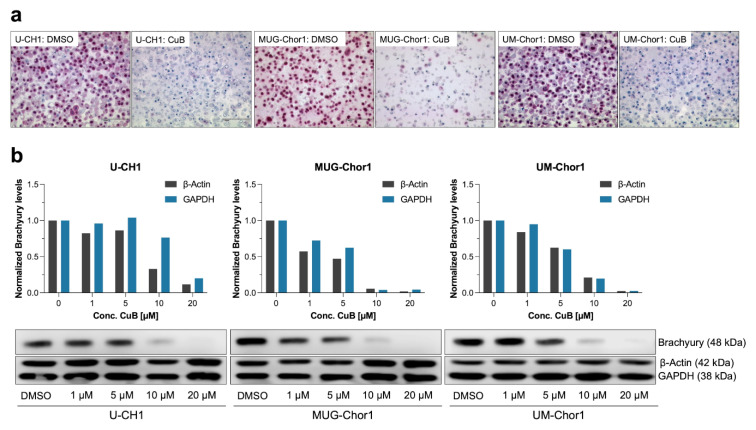
Dose-dependent reduction in brachyury following Cucurbitacin B (CuB) treatment. (**a**) Immunocytochemistry of brachyury in U-CH1, MUG-Chor1, and UM-Chor1 cell lines treated with 20 µM CuB for 48 h, compared to a DMSO control. CuB treatment resulted in a marked reduction in brachyury staining in all cell lines. (**b**) Western blot analysis of brachyury levels in U-CH1, MUG-Chor1, and UM-Chor1 cell lines treated with increasing concentrations of CuB. A clear reduction in brachyury levels was observed at 10 µM CuB in all tested cell lines, with near-complete depletion observed at 20 µM.

Immunocytochemical analysis of CuB-treated (20 µM, 48 h) chordoma cell lines U-CH1, MUG-Chor1, and UM-Chor1 confirmed a dramatic reduction in brachyury levels (Figure 3a).

To determine whether the CuB-induced reduction in brachyury protein levels occurred in a dose-dependent manner, the three chordoma cell lines were treated with increasing concentrations of CuB (1–20 µM) for 48 h, with DMSO-treated cells serving as controls. Western blot analysis demonstrated a dose-dependent decrease in brachyury levels in all cell lines (Figure 3b). In MUG-Chor1 cells, brachyury reduction was detectable at CuB concentrations as low as 1 µM. In UM-Chor1 cells, this effect became evident at 5 µM, while U-CH1 cells exhibited brachyury reduction starting at 10 µM. At 20 µM CuB, brachyury protein was nearly undetectable in all three cell lines.

The differences in sensitivity to CuB-induced brachyury depletion were consistent with IC_50_ values derived from MTS viability assays. MUG-Chor1, with the lowest IC_50_ value (3 µM), exhibited brachyury reduction at the lowest CuB concentration (1 µM). UM-Chor1, with an IC_50_ of 10 µM, displayed brachyury reduction at 5 µM, while U-CH1, with the highest IC_50_ (60 µM), required 10 µM CuB to show similar effects.

These findings indicate that the sensitivity of each cell line to CuB, as reflected by cell viability, corresponds closely to the concentration-dependent loss of brachyury protein.

**Figure 4:**
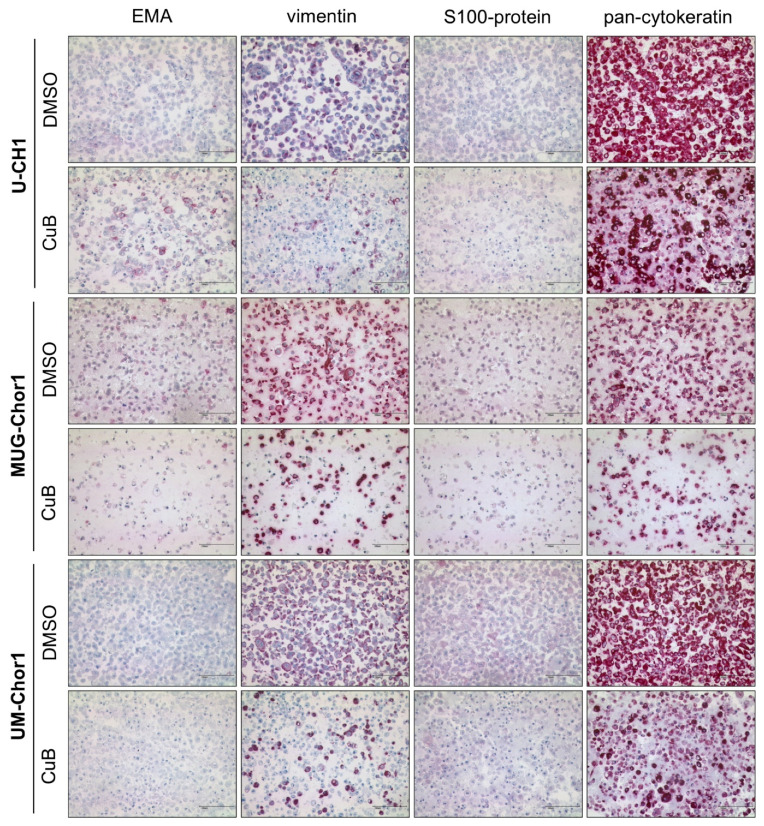
Immunocytochemical analysis of additional chordoma markers. Immunostaining for epithelial membrane antigen (EMA), vimentin, S100-protein, and pan-cytokeratin in U-CH1, MUG-Chor1, and UM-Chor1 treated with 20 µM CuB for 48 h or with DMSO as a control. CuB treatment led to a reduction in vimentin and pan-cytokeratin, while EMA and S100-protein levels remained unchanged. Scale bar = 100 µm.

A reduction in the additional chordoma markers vimentin and pan-cytokeratin was observed by immunocytochemistry, but not as prominent as for brachyury. No alterations in the expression of EMA and S100-protein were observed following CuB treatment in the three chordoma cell lines (Figure 4).

### 2.4. Time-Dependent Effects of Cucurbitacin B on Brachyury Levels

**Figure 5:**
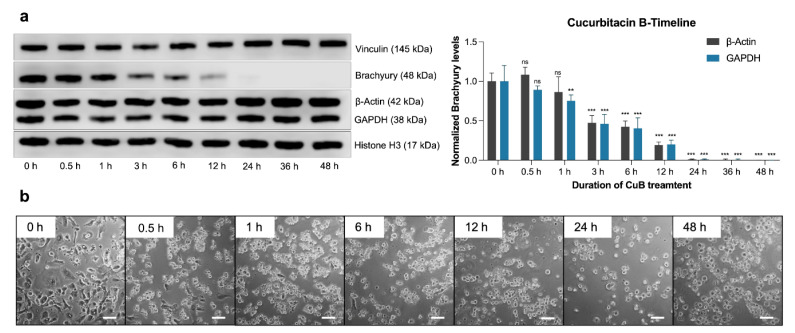
Evaluation of the time dependency of Cucurbitacin B (CuB) treatment in chordoma cells. (**a**) Effect of treatment of U-CH1 cells with 50 µM CuB over a time course of 48 h. Brachyury levels decreased significantly after 3 h and diminished almost completely after 24 h. Error bars represent standard deviation (SD), *n* = 3. Statistical significance: ns = not significant, ** = *p* < 0.01, and *** = *p* < 0.001 (Student’s *t*-test)). (**b**) Morphological changes induced by 50 µM CuB treatment in U-CH1 cells. Cells start to round up and lose adherence after 0.5 h of treatment. Scale bar = 100 µm.

The temporal effects of CuB on brachyury protein levels were assessed in U-CH1 cells treated with 50 µM CuB over a 48 h period. A significant reduction in brachyury expression was detectable as early as 3 h post-treatment, with near-complete depletion observed by 24 h (Figure 5a). Importantly, the expression levels of cytoplasmic housekeeping proteins (β-actin, GAPDH, and vinculin) and nuclear markers (histone H3) remained unchanged throughout the treatment period.

Morphological analysis revealed rapid and dramatic changes in CuB-treated cells (Figure 5b). Within minutes of treatment, cell shape alterations were evident, and by 30 min, most cells had lost adhesion and exhibited a rounded morphology. These changes persisted throughout the 48 h observation period.

The temporal sequence of events indicated that brachyury depletion followed the onset of cytoskeletal alterations. This finding suggests that CuB’s disruption of actin polymerization occurs upstream of brachyury reduction and may be a key driver of the observed protein downregulation. Together, these results highlight the pivotal role of CuB-induced actin polymerization inhibition in initiating downstream effects, including the loss of brachyury.

### 2.5. Transcriptional and Post-Transcriptional Regulation of Brachyury by Cucurbitacin B

**Figure 6:**
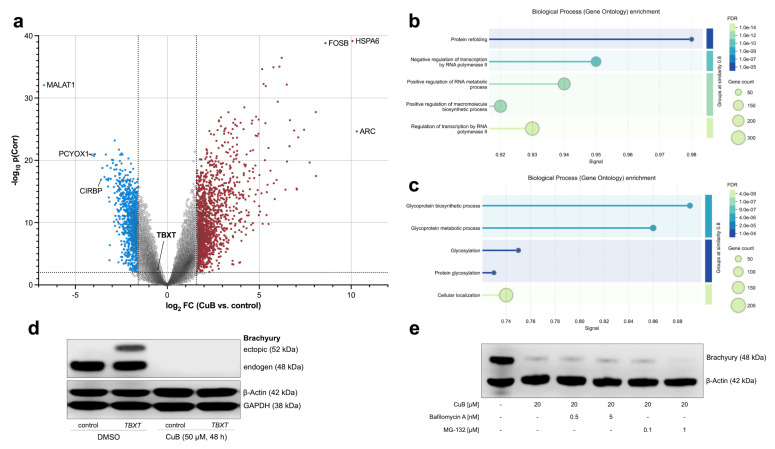
Transcriptional and post-transcriptional regulation of brachyury by Cucurbitacin B (CuB). (**a**) Microarray gene expression analysis of ten chordoma cell lines treated with CuB (50 µM, 48 h) compared to DMSO control, visualized as a volcano plot. The plot shows the log2 fold change (FC) over the corrected −log10 *p*-value, with each gene represented as a dot. The top three downregulated (blue) and upregulated (red) genes, as well as *TBXT* (green), are annotated. Each cell line and condition were analyzed in biological triplicate. (**b**) Top five enriched Gene Ontology (GO) biological processes for significantly downregulated and upregulated (**c**) genes. Dot color represents the false discovery rate (FDR), and dot size corresponds to the gene count within each term. (**d**) Western blot analysis of U-CH1 cells transfected with a myc-tagged open reading frame (ORF) encoding *TBXT* or an empty vector control, followed by treatment with the indicated concentrations of CuB or a DMSO control. Ectopically expressed brachyury, like endogenous brachyury, was lost upon CuB treatment. Experiments were performed in triplicate, and a representative result is shown. (**e**) Western blot analysis of U-CH1 cells co-treated with CuB and either Bafilomycin A or MG-132 at the indicated concentrations. Co-treatments failed to rescue CuB-induced brachyury depletion.

To determine whether CuB regulates brachyury expression at the transcriptional or post-transcriptional level, microarray gene expression analyses were performed on ten chordoma cell lines (U-CH1, U-CH2, U-CH11, U-CH14, U-CH17PII, U-CH19, U-CHCF365, MUG-CC1, MUG-Chor1, and UM-Chor1) treated with CuB (50 µM, 48 h) or DMSO as a vehicle control (Figure 6a). We identified 2273 highly significant differentially expressed genes (p(corr) < 0.01, FC (abs) > 3.0). A full list of all genes is given in Supplementary Table S2 (Additional File S1).

Among these genes, 1481 were upregulated, with *FOSB*, *HSPA6*, and *ARC* being the three most upregulated genes. GO analysis of biological processes revealed high enrichment of terms correlated to protein refolding (FDR 1 × 10^−5^, signal 0.98), negative regulation of transcription by RNA polymerase II (FDR 1 × 10^−9^, signal 0.95), and negative regulation of RNA metabolic processes (FDR 1 × 10^−11^, signal 0.92). Additionally, a strong enrichment of structural constituent of chromatin was observed in the GO category “molecular function” (FDR 1 × 10^−12^, signal 1.5).

A total of 807 genes were downregulated, with *MALAT1*, *PCYOX1* and *CIRBP* being the top three downregulated genes. GO analysis of biological processes revealed an enrichment of terms belonging to protein glycosylation (glycoprotein biosynthetic process, glycoprotein metabolic process, glycosylation and protein glycosylation). The top five terms of the different GO categories for the up- and downregulated genes are depicted in Figure 6b. A full list of the GO terms is given in Supplementary Tables S3 and S4 (Additional File S1). Interestingly, *TBXT* was not amongst the top downregulated genes and exhibited only a modest reduction in expression (FC −1.38; p(corr) = 0.005). The validation of *TBXT* expression in response to CuB treatment was performed in the representative chordoma cell lines U-CH1, MUG-Chor1, and UM-Chor1 by qRT-PCR, confirming a slight transcriptional downregulation of *TBXT* (Supplementary Figure S2; Additional File S2). This implies that the CuB-induced loss of brachyury on the protein level cannot be exclusively explained by transcriptional repression.

To assess whether CuB also regulates brachyury at the post-transcriptional level, U-CH1 cells were transfected with a myc-tagged *TBXT* expression vector, driven by a *TBXT*-promoter-independent mechanism, and subsequently treated with 50 µM CuB for 48 h. Western blot analysis revealed that both endogenous brachyury (detected at ~48 kDa) and ectopically expressed myc-tagged brachyury (detected at ~52 kDa) were depleted following CuB treatment (Figure 6c). This indicates that CuB’s effects on brachyury are essentially not due to transcriptional repression but involve post-transcriptional mechanisms, leading to the depletion of both endogenous and ectopically expressed protein. Interestingly, brachyury degradation could not be rescued using Bafilomycin A (autophagy inhibitor) and MG-132 (proteasome inhibitor; Figure 6d).

## 3. Discussion

During tumor progression, cancer cells undergo significant remodeling of their cytoskeletal architecture, accompanied by decreased cellular stiffness, indicating a potential link between mechanical properties and malignancy. Cellular mechanics are predominantly governed by the cytoskeletal network and its associated proteins. Notably, substantial alterations in cytoskeletal organization are closely tied to malignant transformation and tumor progression including EMT, resulting in distinct changes in both the mechanical properties of tumor cells and their gene expression profiles [28,29].

During embryonic development, brachyury serves as a pivotal regulator of cytoskeletal architecture, influencing the localization of actin fibers [30]. This regulatory function extends to tumorigenesis, where siRNA-mediated knockdown of *TBXT* leads to notable morphological changes and a concurrent downregulation of several cytoskeleton-associated genes, such as *KRT8* and *KRT18* [21,22]. Similarly, in gliomas, brachyury has been shown to regulate the actin cytoskeleton, with reduced expression levels being linked to enhanced cell motility [31]. These findings underscore the critical role of brachyury in maintaining cytoskeletal integrity and highlight its impact on cellular behavior in both developmental and oncological contexts.

The regulatory role of brachyury in cytoskeletal dynamics suggests the intriguing possibility of a reciprocal relationship, where changes in the cytoskeleton might influence brachyury levels. Among various cytoskeleton-disrupting agents, we demonstrated that CuB treatment results in an almost complete loss of brachyury levels, which was accompanied by a reduction in cell viability, as indicated by the MTS assay with IC_50_ values in the micromolar range, consistent with previous observations in other cell lines. However, some cell lines exhibited IC_50_ values in the nanomolar range, suggesting that certain cellular characteristics might contribute to increased sensitivity to CuB [32].

Complementation of LDH cytotoxicity assays revealed approximately 10% of cytotoxic cells at 10 µM CuB, indicating that the reduction in cell viability observed with CuB treatment does not solely stem from direct cell damage, but potentially from additional cellular responses, such as early apoptosis. Notably, CuB has been shown to inhibit ATP citrate lyase (ACLY), an enzyme that plays a crucial role in cellular metabolism by converting citrate into acetyl-CoA, a precursor for both energy production and lipid biosynthesis [33]. This inhibition of ACLY may have indirect consequences on both the MTS and LDH assays, potentially skewing their results.

Interestingly, although LatB induced similar morphological changes, it did not affect brachyury expression. Both compounds disrupt the cytoskeleton by promoting F-actin depolymerization. However, the effect of LatB is known to be reversible, as we observed in chordoma cell line U-CH1. In contrast, CuB causes irreversible aggregation of G-actin [27].

Consistent with reports from Wakimoto et al. and Yin et al., which documented rapid morphological changes and cytoskeletal disruption in breast cancer and glioblastoma cells associated with F-actin disassembly, our findings reveal that chordoma cells detach and round within minutes of CuB treatment [34,35]. Moreover, we demonstrate that these morphological changes in chordoma cells are accompanied by an almost complete loss of brachyury. However, the mechanism underlying the reduction in brachyury levels remains unclear. Our data suggest that the loss of brachyury is both dose- and time-dependent. Depending on the chordoma cell line, an initial reduction in brachyury levels was observed at 1 µM CuB, with complete degradation occurring at 20 µM in all tested cell lines. Time course analyses revealed that morphological changes in the cells preceded the reduction in brachyury levels, suggesting that the loss of brachyury is a direct or indirect consequence of actin cytoskeleton disruption.

Recent studies have identified mechanisms by which actin dynamics directly affect gene transcription [29]. For instance, during adipogenesis, monomeric G-actin binds to the transcriptional co-activator MKL1, preventing its nuclear translocation and hindering the induction of the adipocytic differentiation program [36]. Additionally, disruption of the cytoplasmic actin cytoskeleton is transmitted to the nucleoskeleton via Linker of Nucleus and Cytoskeleton (LINC) protein connections, enabling direct alterations in nuclear shape, transcription factor entry, and chromatin architecture [37].

By microarray gene expression analysis, we have observed profound alterations in global gene expression, with most genes being upregulated in response to CuB treatment. We show that CuB-induced changes in gene expression do not consist of a general downregulation, but rather in a specific modulation of transcriptional activity.

Among the upregulated genes, we observed a strong enrichment for the GO term *protein refolding*, suggesting that irreversible disruption of the actin cytoskeleton by CuB might lead to the accumulation of misfolded or aggregated proteins, which in turn may trigger the activation of protein refolding mechanisms [38]. In various solid tumors, CuB, along with structurally similar compounds such as Cucurbitacin I and Cucurbitacin E, has been shown to induce endoplasmic reticulum (ER) stress, a condition closely linked to the activation of the unfolded protein response (UPR) [39,40,41]. By impairing proteostasis and increasing the burden on the ER’s protein folding machinery, CuB might exacerbate cellular stress, thereby inducing brachyury degradation and apoptosis in chordoma cells.

*ARC* (Activity-Regulated Cytoskeleton-Associated Protein) was the most upregulated gene. In the context of the cytoskeleton, ARC is involved in actin filament dynamics, which are essential for cellular responses to various stimuli, including growth signals and stress. Additionally, it has been shown to regulate actin dynamics, impacting cellular processes such as migration, shape changes, and motility [42]. This suggests that ARC may facilitate the reorganization of the cytoskeleton in response to CuB-induced disruptions, thereby contributing to cellular adaptation to stress. This upregulation could reflect an attempt by the cell to restore homeostasis by modulating actin filament dynamics and preventing the accumulation of misfolded proteins.

The enriched GO terms of the downregulated genes were associated with glycosylation and glycoprotein biosynthesis. N-linked glycosylation within the ER is essential for monitoring the folding state of proteins [43]. The glycosylation status within the ER determines whether proteins are directed to the Golgi apparatus for further processing or targeted for degradation via the ER-associated degradation pathway. The accumulation of unfolded or misfolded glycoproteins in the ER lumen triggers the activation of the UPR, a stress-signaling pathway that promotes the degradation of defective proteins [44].

Consequently, the CuB-induced degradation of brachyury and apoptosis in chordoma cells might involve a mechanism in which irreversible disruption of the actin cytoskeleton leads to protein misfolding, ER stress, and ultimately cell death. *TBXT*-ORF experiments further support the hypothesis of a post-transcriptional knockdown of brachyury. The degradation of brachyury protein may, in turn, disrupt the auto-regulatory enhancer loop of brachyury, potentially explaining the slight downregulation of *TBXT* mRNA expression that we observed [21]. Notably, CuB also modulates several additional pathways implicated in chordomagenesis, including the WNT/β-catenin, MAPK, and JAK2/STAT3 pathways, which may contribute to the downregulation of brachyury and the induction of apoptosis [45,46,47].

The inability to rescue brachyury degradation using the proteasome inhibitor MG-132 or the autophagy inhibitor Bafilomycin A indicates the involvement of alternative degradation pathways which remain to be elucidated. Consequently, additional studies are required to validate this mechanistic framework. Although brachyury is known to contain six N-glycosylation sites, it also remains unclear why it is selectively targeted for degradation while other proteins remain unaffected [48]. Intriguingly, previous studies have shown that Afatinib, initially developed as an EGFR inhibitor, also induces brachyury degradation, pointing to a unique vulnerability of brachyury to specific degradation mechanisms [10].

Our data indicate that brachyury is effectively targeted through irreversible disruption of the actin cytoskeleton by CuB. However, the observation that only the irreversible inhibition of the actin cytoskeleton by CuB, but not the reversible inhibition by LatB, affects brachyury suggests that the inhibition of the cytoskeleton itself may not directly impact brachyury. Instead, cellular consequences such as ER stress and protein refolding mechanisms are more likely to exert an indirect influence on brachyury. Although treatment with CuB leads to a significant reduction in chordoma cell viability, induction of apoptosis, and undetectable levels of brachyury, supporting the promising therapeutic potential of brachyury degradation, its non-selective toxicity should also result in adverse effects in normal cells [32]. While CuB itself might not be compatible for clinical applications, it can serve as chemical lead for more selective compounds. Given brachyury’s essential role in the survival and proliferation of chordoma cells, targeted degradation of this protein represents a compelling therapeutic strategy that needs to be further explored [49].

## 4. Material and Methods

### 4.1. Cell Culture

Chordoma cell lines were maintained in a 4:1 mixture of Iscove’s Modified Dulbecco’s Medium (IMDM) and RPMI 1640 (Lonza, Basel, Switzerland), supplemented with 10% fetal bovine serum (Biochrom AG, Berlin, Germany), 2 mM L-glutamine, and penicillin/streptomycin, as previously described [24]. The chordoma cell lines U-CH1, U-CH2, U-CH11, U-CH14, U-CH17PII, and U-CH19 were established at the Institute of Pathology, University of Ulm, as previously reported in the literature [2,16,50]. The U-CHCF365 cell line was established at the Institute of Pathology, University of Ulm, from a chordoma xenograft provided by the Chordoma Foundation (www.chordomafoundation.org, accessed on 17 April 2025). The UM-Chor1 and MUG-Chor1 cell lines were both provided by the Chordoma Foundation, while the MUG-CC1 cell line was provided by Beate Rinner [51].

Informed written consent was obtained from all patients, and the study was conducted in accordance with the Declaration of Helsinki and approved by the local ethics committee (votes 369/17 and 373/17).

Cell lines underwent quality control through short tandem repeat (STR) analysis using the GenomeLab STR Primer Set Kit (Beckman Coulter, Krefeld, Germany) and were regularly tested for mycoplasma contamination to ensure their integrity. Cell morphology was captured using the PAULA cell imager (Leica Microsystems, Wetzlar, Germany).

### 4.2. Reagents

The following reagents were used in this study: Cucurbitacin B hydrate (Sigma-Aldrich, St. Louis, MO, USA), Latrunculin B (MedChem Express, Monmouth Junction, NJ, USA), Vinblastine sulfate (Selleckchem, Houston, TX, USA), Colchicine (Selleckchem), and Narciclasine (Sigma-Aldrich). All compounds, except for Colchicine, were dissolved in dimethyl sulfoxide (DMSO (Sigma Aldrich, St. Louis, MO, USA)) at the appropriate concentrations. Colchicine was dissolved in ddH_2_O. Stock solutions were prepared according to the manufacturer’s instructions and stored at −20 °C until use.

### 4.3. Cell Viability Assay

The impact of Cucurbitacin B (CuB) on cell viability was evaluated using the colorimetric MTS cell proliferation assay kit (Abcam, Cambridge, UK), according to the manufacturer’s instructions. The MTS assay measures the metabolic activity of viable cells based on the reduction of MTS tetrazolium salt to formazan by NAD(P)H-dependent dehydrogenase enzymes in metabolically active cells.

In brief, U-CH1, MUG-Chor1, and UM-Chor1 chordoma cells were seeded at a density of 5000 cells per well in a 96-well plate and allowed to adhere overnight. Afterward, the cells were incubated with increasing concentrations of CuB (0.001–75 µM) for 48 h in biological and technical triplicate. IC_50_ values were determined using GraphPad Prism v10 (GraphPad Software Inc., Boston, MA, USA). The absorbance was measured at 490 nm and 650 nm using a microplate reader.

### 4.4. Assessment of Cytotoxic Effects of Cucurbitacin B

Cytotoxic effects of CuB were measured using the Lactate Dehydrogenase (LDH) Cytotoxicity Assay Kit (Pierce, ThermoFisher Scientific, Grand Island, NY, USA) following the manufacturer’s protocol. The LDH assay measures the release of lactate dehydrogenase (LDH) from damaged (necrotic and late apoptotic) cells, which correlates with cytotoxicity.

In brief, chordoma cells (5 × 10^3^ cells/well) were seeded in 96-well plates and allowed to adhere overnight, followed by incubation with CuB for 48 h. Next, 50 µL of culture supernatant was collected and incubated with 50 μL of the LDH reaction mixture for 30 min in a 96-well plate. Finally, 50 μL of LDH stop solution was added to stop the reaction. The absorbance was measured at 490 nm and 680 nm using a microplate reader.

### 4.5. Immunostaining of Formalin-Fixed and Paraffin-Embedded Cell Blocks

Immunostaining of formalin-fixed and paraffin-embedded cell blocks was performed using the avidin–biotin complex method with the K005 AP/RED Detection System (Dako, Glostrup, Denmark). The primary antibodies utilized included cleaved caspase-3 (Asp175; 1:100; Cell Signaling Technology, Danvers, MA, USA), brachyury (EPR18113; 1:4000; Abcam, Cambridge, UK), EMA (E29; 1:500; Dako), S100-protein (Z0311; 1:500; Dako), vimentin (VIM3B4; 1:300; Dako), and pan-cytokeratin (AE1 + AE3; 1:100; Dako).

### 4.6. Western Blot Analysis

Western blotting was performed as previously described, with minor adaptations [2]. In brief, proteins were separated by SDS-PAGE and transferred to a nitrocellulose membrane. The membrane was blocked with 3% BSA for 60 min, followed by incubation with the primary antibody at 4 °C overnight. An additional blocking step for 30 min with 10% skim milk was performed prior to incubation of an appropriate secondary antibody (goat anti-mouse IgG (H + L) 1:10,000, ThermoFisher Scientific; goat anti-rabbit IgG (whole molecule) 1:2000, Sigma-Aldrich). The WesternSure Chemiluminescent Substrate (LI-CORE Biosciences, Lincoln, NE, USA) was applied for detection. Immunoblots were quantified by densitometry using the ImageJ software (v1.52e; NIH). The following primary antibodies were used: brachyury (D2Z3J; 1:2000; Cell Signaling Technology), GAPDH (14C10; 1:2000; Cell Signaling Technology), β-actin (BA3R; 1:2000; ThermoFisher Scientific), vinculin (H-10; 1:1000; Santa Cruz, Dallas, TX, USA), and histone H3 (polyclonal; 1:2000; Cell Signaling).

At least both β-actin and GAPDH were used as housekeeping proteins in all Western blots to ensure the reliability of our results. Given that cytoskeletal disruption could potentially influence actin levels, the inclusion of GAPDH provided an additional reference to confirm that the observed changes in protein expression were not due to variations in actin expression.

### 4.7. Assessment of Time- and Dose-Dependency of Cucurbitacin B-Induced Brachyury Depletion

To evaluate the kinetics of CuB-induced brachyury depletion, U-CH1 cells were incubated with either 50 µM CuB or an appropriate vehicle control (0.5% dimethyl sulfoxide, DMSO) at 37 °C in a 5% CO_2_ atmosphere. Total protein was harvested at various time points 0 h–48 h post-treatment to assess the temporal dynamics of brachyury levels. In parallel, to investigate the dose-dependency of CuB-induced brachyury depletion, U-CH1, MUG-Chor1, and UM-Chor1 cell lines were treated with a range of CuB concentrations: 0 µM (0.5% DMSO), 1 µM, 5 µM, 10 µM, and 20 µM. Following a 48 h incubation, total protein was isolated from the cells. Brachyury protein levels were subsequently quantified using Western blot analysis, as previously described.

### 4.8. TBXT Open Reading Frame (ORF) Rescue Experiment

U-CH1 cells were plated in 6-well plates at a density of 5 × 10^5^ cells per well. The following day, the cells were transfected with 2 µg of the pCMV6 expression vector encoding *TBXT* or the corresponding empty backbone control vector (both obtained from OriGene, Rockville, MD, USA) using 10 µL of Lipofectamine 2000 (ThermoFisher Scientific) transfection reagent. Two days post-transfection, the cells were treated with either 50 µM CuB or an equivalent volume of dimethyl sulfoxide (DMSO) as a control. Protein extraction was performed 24 h after the addition of the compound or DMSO, and the samples were subsequently analyzed using Western blotting.

### 4.9. Inhibition of Autophagy and Proteasome System

U-CH1 cells were seeded into 6-well plates at a density of 5 × 10^5^ cells per well and treated with CuB at a final concentration of 50 µM. To inhibit autophagic activity, cells were co-treated with Bafilomycin A (Santa Cruz, Dallas, TX, USA) at concentrations of 0.5 nM and 5 nM for 48 h. Proteasomal system inhibition was achieved by applying MG-132 (Selleckchem) at concentrations of 0.1 µM and 1 µM for the same duration. Following treatment, brachyury protein levels were assessed using Western blot analysis to evaluate the effects of autophagy and proteasomal inhibition on CuB-treated cells.

### 4.10. RNA Isolation and cDNA Synthesis from Compound-Treated Cells

Three chordoma cell lines, U-CH1, MUG-Chor1, and UM-Chor1, were cultured in 6-well plates at a density of 500,000 cells per well, allowing for overnight adherence. The following day, the culture media was replaced with standard growth media supplemented with either 50 µM CuB or an equivalent volume of DMSO as a control. After 48 h of treatment, both compound-treated and DMSO-treated cells were harvested for analysis.

Total RNA was isolated from the chordoma cell lines using the Qiagen RNeasy Kit (Qiagen, Hilden, Germany), following the manufacturer’s protocol to ensure optimal RNA integrity and yield. The isolated RNA was then subjected to cDNA synthesis utilizing the SuperScript IV Reverse Transcriptase Kit (ThermoFisher Scientific). To ensure statistical robustness, three biological replicates were prepared for each experimental condition.

### 4.11. Reverse Transcription Quantitative Polymerase Chain Reaction (qRT-PCR)

The Quantitect SybrGreen Kit (Qiagen) was used according to the manufacturer’s instructions. The relative expression levels of *TBXT* were calculated using the ΔΔCT method, with *GAPDH* serving as the housekeeping gene for normalization. The primer sequences and their respective annealing temperatures used for amplification are provided in Supplementary Table S1 (Additional File S1).

### 4.12. Microarray Gene Expression Analysis

Microarray gene expression profiling was conducted as previously described [2]. Raw data were processed and analyzed using GeneSpring version 14.9 software, with statistical significance defined at *p* < 0.01 and an absolute fold change (FC) > 3. Ten chordoma cell lines, specifically U-CH1, U-CH2, U-CH11, U-CH14, U-CH17PII, U-CH19, U-CHCF365, MUG-CC1, MUG-Chor1, and UM-Chor1, were treated for 48 h with 50 µM CuB or an equivalent volume of DMSO as a control. All conditions and cell lines were tested in biological triplicate.

### 4.13. GO-Term Analysis

The STRING database v.12 built-in enrichment analysis tool was used to identify overrepresented Gene Ontology (GO) terms within the input protein set [52]. Interactions were filtered to include only high-confidence associations with a minimum confidence score of 0.7. Active interaction sources were restricted to experimental data and co-expression.

### 4.14. Statistical Analysis

For statistical analyses, Student’s *t*-tests were performed. A *p*-value ≤ 0.05 was considered to define significant differences.

## 5. Conclusions

In conclusion, this study highlights the critical role of actin cytoskeleton integrity in maintaining brachyury stability in chordoma cells. By demonstrating that CuB-induced actin cytoskeleton disruption leads to brachyury depletion through mechanisms involving protein refolding dysfunction and ER stress, rather than transcriptional repression, these findings open new avenues for therapeutic intervention. Targeting actin cytoskeleton dynamics or the pathways regulating protein folding and stress responses could offer promising strategies for the development of novel treatments for chordoma, addressing an urgent need for more effective therapies for this challenging malignancy.

## Data Availability

The data generated in this study are available upon request from the corresponding author.

## Supplementary Materials

The following supporting information can be downloaded at: https://www.mdpi.com/article/10.3390/ijms26083864/s1.

## Abbreviations

ARC	Activity-Regulated Cytoskeleton-Associated Protein
cDNA	Complementary DNA
Colch.	Colchicine
CuB	Cucurbitacin B
ddH_2_O	Double-distilled water
DMSO	Dimethyl Sulfoxide
EMA	Epithelial Membrane Antigen
ER	Endoplasmic Reticulum
FDR	False Discovery Rate
GAPDH	Glyceraldehyde-3-Phosphate Dehydrogenase
GO	Gene Ontology
HSPA6	Heat Shock Protein Family A Member 6
IC_50_	Half Maximal Inhibitory Concentration
JAK2	Janus Kinase 2
LatB	Latrunculin B
LDH	Lactate Dehydrogenase
MALAT1	Metastasis Associated Lung Adenocarcinoma Transcript 1
MAPK	Mitogen-Activated Protein Kinase
MTS	3-(4,5-dimethylthiazol-2-yl)-5-(3-carboxymethoxyphenyl)-2-(4- sulfophenyl)-2H-tetrazolium
Narcil.	Narciclasine
ORF	Open Reading Frame
qRT-PCR	Quantitative Reverse Transcription Polymerase Chain Reaction
Rho/ROCK	Rho-associated coiled-coil kinase pathway
STAT3	Signal Transducer and Activator of Transcription 3
TBXT	T-Box Transcription Factor
UPR	Unfolded Protein Response
Vinblast.	Vinblastine
