# Supplementary material for: Cucurbitacin B Exhibits Antitumor Effects on Chordoma Cells via Disruption of Brachyury
Source: Int J Mol Sci. 2025 Apr 18;26(8):3864. doi: 10.3390/ijms26083864 (PMC12028342; doi:10.3390/ijms26083864)
Supplement: Supplementary file 1 [file ijms-26-03864-s001.zip › Additional file S2.pdf]

Supplementary Figures

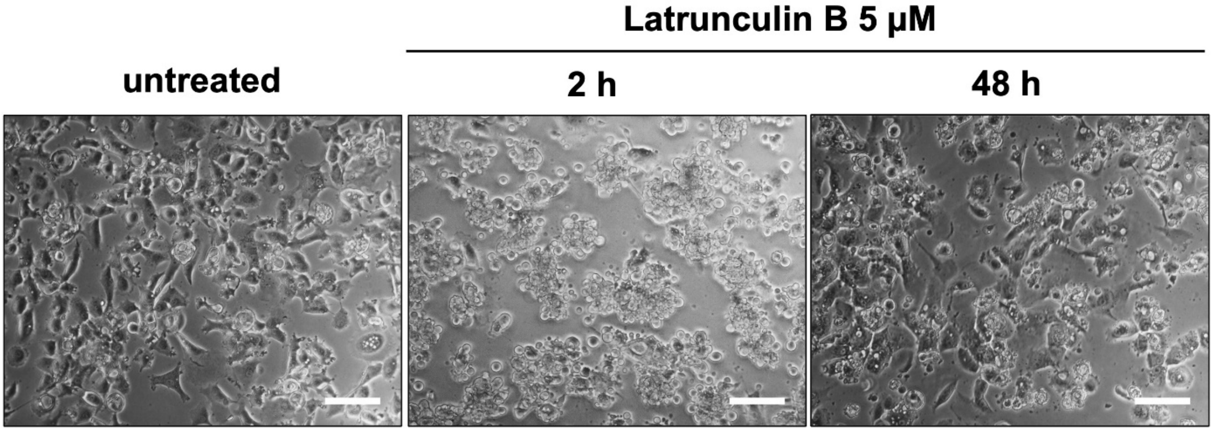

Suppl. Figure S1: U-CH1 chordoma cells begin to re-adhere following 48 hours of Latrunculin B (LatB) treatment. Phase-contrast microscopy images of U-CH1 cells exposed to LatB for two hours or 48 hours. After two hours of treatment, cells appear rounded and have lost adherence to the substrate. By 48 hours, cells have re-adhered and exhibit a morphology similar to that of untreated U-CH1 cells. Scale bar = 100 µm.

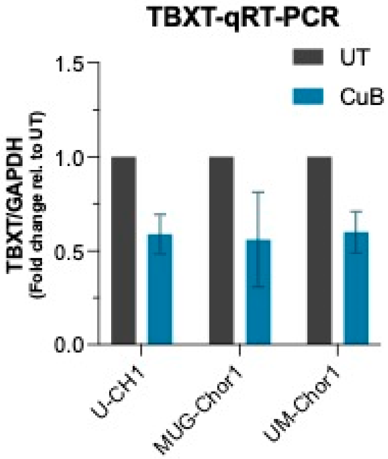

Suppl. Figure S2: *TBXT* mRNA expression levels following Cucurbitacin B (CuB) treatment. Chordoma cell lines U-CH1, MUG-Chor1, and UM-Chor1 were treated with 50 µM CuB for 48 hours. *TBXT* expression was quantified using qRT-PCR and normalized to GAPDH expression. Data are presented as fold change relative to untreated (UT) cells. Error bars indicate standard deviation (SD), with experiments performed in biological duplicates (n = 2).
